# The Impact of Recent Tobacco Regulations and COVID-19 Restrictions and Implications for Future E-Cigarette Retail: Perspectives from Vape and Vape-and-Smoke Shop Merchants

**DOI:** 10.3390/ijerph19073855

**Published:** 2022-03-24

**Authors:** Zongshuan Duan, Katelyn F. Romm, Lisa Henriksen, Nina C. Schleicher, Trent O. Johnson, Theodore L. Wagener, Steven Y. Sussman, Barbara A. Schillo, Jidong Huang, Carla J. Berg

**Affiliations:** 1 Department of Prevention and Community Health, Milken Institute School of Public Health, George Washington University, Washington, DC 20052, USA; kromm@gwu.edu (K.F.R.); carlaberg@gwu.edu (C.J.B.); 2 Stanford Prevention Research Center, Department of Medicine, Stanford University School of Medicine, Palo Alto, CA 94304, USA; lhenriksen@stanford.edu (L.H.); ninasch@stanford.edu (N.C.S.); trentj@stanford.edu (T.O.J.); 3 Center for Tobacco Research, Ohio State University Comprehensive Cancer Center, Columbus, OH 43210, USA; theodore.wagener@osumc.edu; 4 Department of Population and Public Health Sciences, Keck School of Medicine, University of Southern California, Los Angeles, CA 90032, USA; ssussma@usc.edu; 5 Truth Initiative Schroeder Institute, Washington, DC 20001, USA; bschillo@truthinitiative.org; 6 Department of Health Policy & Behavioral Sciences, School of Public Health, Georgia State University, Atlanta, GA 30303, USA; jhuang17@gsu.edu; 7 George Washington Cancer Center, George Washington University, Washington, DC 20052, USA

**Keywords:** tobacco regulations, Tobacco 21, flavored e-cigarette ban, COVID-19 orders, vape shop, vape-and-smoke shop, retail activities

## Abstract

Background: Tobacco regulations and COVID-19 state orders have substantially impacted vape retail. This study assessed vape retailers’ perspectives regarding regulations and future retail activities. Methods: In March–June 2021, 60 owners or managers of vape or vape-and-smoke shops (*n* = 34 vs. *n* = 26) in six US metropolitan areas completed an online survey assessing: (1) current and future promotional strategies and product offerings; and (2) experiences with federal minimum legal sales age (T21) policies, the federal flavored e-cigarette ban, and COVID-19-related orders. Quantitative data were analyzed descriptively; qualitative responses to open-ended questions were thematically analyzed. Results: Most participants had websites (65.0%), used social media for promotion (71.7%), offered curbside pickup (51.7%), and sold CBD (e.g., 73.3% vape products, 80.0% other); many also sold other tobacco products. Knowledge varied regarding state/local policies in effect before federal policies. Participants perceived tobacco regulations and COVID-19 orders as somewhat easy to understand/implement and perceived noncompliance consequences as somewhat severe. Qualitative themes indicated concerns regarding regulations’ negative impacts (e.g., sales/customer loss, customers switching to combustibles), insufficient evidence base, challenges explaining regulations to customers, and concerns about future regulatory actions. Conclusions: Surveillance of tobacco retail, consumer behavior, and regulatory compliance is warranted as policies regarding nicotine and cannabis continue evolving.

## 1. Introduction

Electronic cigarettes (i.e., e-cigarettes or vape products) have emerged globally and in the US with substantial public health impacts [1]. Vape retailers are important sources of information regarding e-cigarettes for customers [2,3], potentially impacting their vaping behaviors and perceptions of tobacco products [4,5]. Notably, prominent sources of e-cigarettes are tobacco specialty stores (i.e., “vape shops” that exclusively sell e-cigarettes [6] or “smoke shops” that may sell vape and other tobacco products [7]); such retailers accounted for approximately 19% of retail e-cigarette sales in 2019 [8]. Understanding merchants’ current and future retail activities and perspectives regarding future regulations may have important implications for regulatory efforts. Doing so is particularly critical during pivotal regulatory and societal changes in the tobacco product marketplace in the US.

In the US, the regulation of e-cigarettes has dramatically evolved in the past 5 years. In 2016, the Deeming Rule extended the US Food and Drug Administration’s (FDA) authority to e-cigarettes, including manufacturing, retail, and marketing (e.g., prohibiting free samples, false or misleading ads) [9]. Other federal, state, and local regulations that have impacted tobacco retail have also been advanced or gone into effect, including those restricting e-cigarette sales and distribution. In January 2020, the FDA announced a ban on all flavors in cartridge (pod-based) e-cigarettes typically sold in vape shops; notably, this ban does not include tobacco and menthol flavors and also does not extend to other types of e-cigarettes (e.g., disposables, mod systems) [10]. Due to the exemptions in the federal ban, state and local restrictions on flavored e-cigarettes are closing important loopholes [11]. As of October 2021, 5 states and 338 localities have implemented more comprehensive restrictions on flavors, with state and local sales restrictions on flavored e-cigarettes likely to continue to expand [12,13]. Moreover, the Prevent All Cigarette Trafficking (PACT) Act (which prohibits USPS delivery of cigarettes and smokeless tobacco to consumers) was expanded to include e-cigarettes in December 2020 [14].

Impacting tobacco retail more broadly, federal legislation raised the minimum legal sales age for e-cigarette sales from 18 to 21 (i.e., Tobacco 21 [T21]) in December 2019 [15]. Despite evidence of the public health benefits from T21 (e.g., youth prevention) [16,17], and the potential to increase awareness and support for tobacco control efforts among retailers and consumers [18], there are high noncompliance rates in some states [19,20], noncompliance may be underestimated [19], and gaps in FDA enforcement protocols have been identified [20,21]. In addition, there is growing concern about noncompliance rates for e-cigarette sales, especially among tobacco specialty shops [22,23], and online retailers [22,24]. In the future, federal legislation likely to advance includes: expanding flavored tobacco product restrictions [25], implementing pictorial health warning labels (in 2022) [26], and increasing federal tobacco taxes [27]. These initiatives could lead to changes in tobacco and e-cigarette use (e.g., quitting, product substitution) [28], as well as changes at the point-of-sale (e.g., price discounts) [29,30].

COVID-19 has also had substantial impacts on e-cigarette retail and consumers. For example, many retailers adapted to the pandemic by conducting business online, and many consumers now purchase goods online and use delivery services [31]. Although many state governors issued executive orders requiring the temporary closure of tobacco specialty shops during shelter-in-place periods, compliance with the mandated closures was low among some vape shops, with considerable variations across states and localities and by neighborhood demographics [32,33].

As the nicotine marketplace has evolved and key regulations have gone into effect that may substantially impact the vape retail environment and product use [34], understanding merchants’ perspectives regarding such regulations may have important implications for regulatory efforts [35]. For example, prior research involving vape retail merchants has provided unique insights into their marketing strategies [36,37,38], as well as into their concerns that policies are not distinguishing e-cigarettes from traditional tobacco (e.g., cigarettes) [39,40,41] and/or not taking into account the impact on the potential harm reduction that could result from using e-cigarettes [37,38,40]. As these more recent policies and regulatory changes (e.g., flavor restrictions, T21, COVID-19-related orders) have occurred, engaging the perspectives of vape retailers may provide unique insights into the future of vape products and retail. Therefore, this study analyzed data from online surveys of 60 owners or managers of vape and vape-and-smoke shops across six US metropolitan statistical areas (MSAs) conducted in March–June 2021. Specifically, we examined: (1) current marketing strategies; (2) perspectives regarding the impact of federal T21 law, the federal flavored e-cigarette ban, and COVID-19-related state orders; and (3) anticipated future retail activities.

## 2. Materials and Methods

### 2.1. Participants and Procedures

The parent study, detailed elsewhere [23,34,42,43,44], was approved by the Emory University Institutional Review Board. This study focuses on 6 MSAs (Atlanta, Georgia; Boston, Massachusetts; Minneapolis, Minnesota; Oklahoma City, Oklahoma; San Diego, California; Seattle, Washington), selected based on the differences in state policy and retail markets for tobacco and cannabis. Relevant to this study, before the federal T21 law went into effect (20 December 2019), among the 19 states that enacted state T21 policies were California (enacted 4 May 2016; effective 9 June 2016), Massachusetts (enacted 27 July 2018; effective 31 December 2018), and Washington (enacted 5 April 2019; effective 1 January 2020)—but not Georgia, Minnesota, and Oklahoma [45]. T21 laws had also gone into effect in several localities, including Boston (effective 15 February 2016), Minneapolis (effective 1 October 2018), and others in these MSAs—but not in the Atlanta, Oklahoma City, Seattle, or San Diego MSAs (although other localities in California had) [45]. In November 2019, Massachusetts became the first state to restrict the sale of all flavored tobacco products (effective 27 November 2019 for e-cigarettes; 1 June 2020 for other tobacco products). Washington had an emergency rule to temporarily restrict flavored e-cigarette sales in the fall of 2019 (effective 9 October 2019–February 2020) [46]. Such local laws have been implemented in several localities in California, Massachusetts, and Minnesota, but not in Georgia, Oklahoma, or Washington [46].

The current study analyzes data from surveys of owners or managers conducted in May–September 2021. To provide the sampling frame for this survey study, first, we conducted application program interface (API) queries (using HTTP requests to access and use data) via Yelp Graph QL for stores classified as “vape shops,” and Google Maps for stores classified as “vaporizer store”, “vape shop”, and “vape store” [32,34,44], as conducted in our prior research [34]. After an initial round of data cleaning and geocoding the stores to the 6 MSAs, phone calls (*n* = 1718) were made in May–September 2021 to verify that retailers sold e-cigarettes (i.e., devices and/or e-liquids) and were open for business [32,34,44]. Completed phone calls of open vape retailers (*n* = 965, completion rate = 56.2%) culminated with invitations to owners and managers to complete a self-administered online survey for an incentive of a USD 35 Amazon e-gift card. Of the 965 retailers, 339 owners/managers declined receiving a survey link via email or text, and 447 were not able to be reached in the course of the phone verifications (e.g., research staff only spoke with vape retail staff), resulting in 179 survey links being emailed or texted to owners or managers. Of the 94 (52.5%) owners and managers who answered the questionnaire, analyses were restricted to the 60 (63.8%) who provided complete data, with the remaining 34 partial completions largely providing answers to only the first section (or couple of items) of the survey. Thus, analyses focus on the 60 surveys with complete responses.

### 2.2. Measures

Measures were adapted from existing literature that has assessed point-of-sale marketing for tobacco [47], including such marketing specific to vape retail [23] and to regulatory and COVID-19 impact on tobacco retailers [35,47].

*Participant characteristics* included: position at the store, age, gender, and race/ethnicity. *Store characteristics* included: state/MSA, type of store (i.e., vape-only or vape-and-smoke shop), if part of a chain, and if in business at this location in June 2020.

We assessed current *promotional activities* (i.e., have a website, online sales, home delivery, curbside pickup, social media for promotion) and *product offerings* (i.e., own brand of nicotine e-liquid, nicotine concentrates for adding to zero-nicotine flavored e-liquid, other tobacco products, CBD or cannabis products, etc.; see Table 1 for full list).

To assess *knowledge of current tobacco regulations in their community*, we asked whether their retail location had a state or local: (1) T21 policy in place before federal T21; and (2) e-cigarette flavor restrictions before federal restrictions on flavored cartridge-based e-cigarettes. To assess *perceived difficulty understanding and implementing tobacco regulations*, we asked: “How easy or difficult has it been for you to: understand the federal T21 legislation? follow the federal T21 legislation in your shop? understand the federal flavored e-cigarette ban? follow the federal flavored e-cigarette ban?” (1 = “Very easy”, 2 = “Somewhat easy”, 3 = “Do not know”, 4 = “Somewhat difficult”, 5 = “Very difficult”). Open-ended questions included: (1) “What was or has been difficult about understanding and/or following the T21 legislation? The flavored product bans?” and (2) “In your experience, what is being done to enforce or oversee compliance with the flavored e-cigarette ban? What do you perceive as the consequences or risks of not being compliant?” To assess *perceived consequences of noncompliance to tobacco regulations*, we asked about agreement with: “the consequences for violating: (a) the T21 policy… and (b) the flavored e-cigarette ban… are severe” (1 = “Strongly disagree”, 2 = “Somewhat disagree”, 3 = “Neutral, Do not know”, 4 = “Somewhat agree”, 5 = “Strongly agree”). Open-ended questions asked: “In your experience, what is being done to enforce or oversee compliance with the federal T21 laws and the e-cigarette flavor ban? What do you perceive as the consequences or risks of not being compliant?”

To assess *perceived difficulty understanding and implementing COVID-19 orders*, we asked: “During April–June 2020 COVID-related restrictions on business operations, how easy or difficult was it to: understand the COVID-19-related restrictions on business operations? follow the COVID-19-related restrictions on business operations?” (1 = “Very easy”, 2 = “Somewhat easy”, 3 = “Do not know”, 4 = “Somewhat difficult”, 5 = “Very difficult”). Open-ended question asked: “What was or has been difficult about understanding and/or following the COVID-related policies?”.

*Impact of tobacco regulations and COVID-19 on retail* was assessed by asking: “To what extent did COVID-19 and tobacco regulation impact: device sales, e-liquid sales, device prices, e-liquid prices, number of device types sold, number of e-liquid flavors/types sold, number of customers who are frequent patrons (“usuals”), number of customers who are not frequent patrons, employee retention, and online sales?” (1 = “Decreased a lot”, 2 = “Somewhat decreased”, 3 = “No change”, 4 = “Somewhat increased”, 5 = “Increased a lot”). Open-ended questions included: (1) “How have your customers been impacted by the federal T21 legislation? The flavored e-cigarette ban?” (2) “Outside of T21 and the flavor ban, what other tobacco-related regulations are impacting your shop or do you think will impact your shop in the future?” and (3) “How have your vendors responded to COVID-19?”

**Figure 1:**
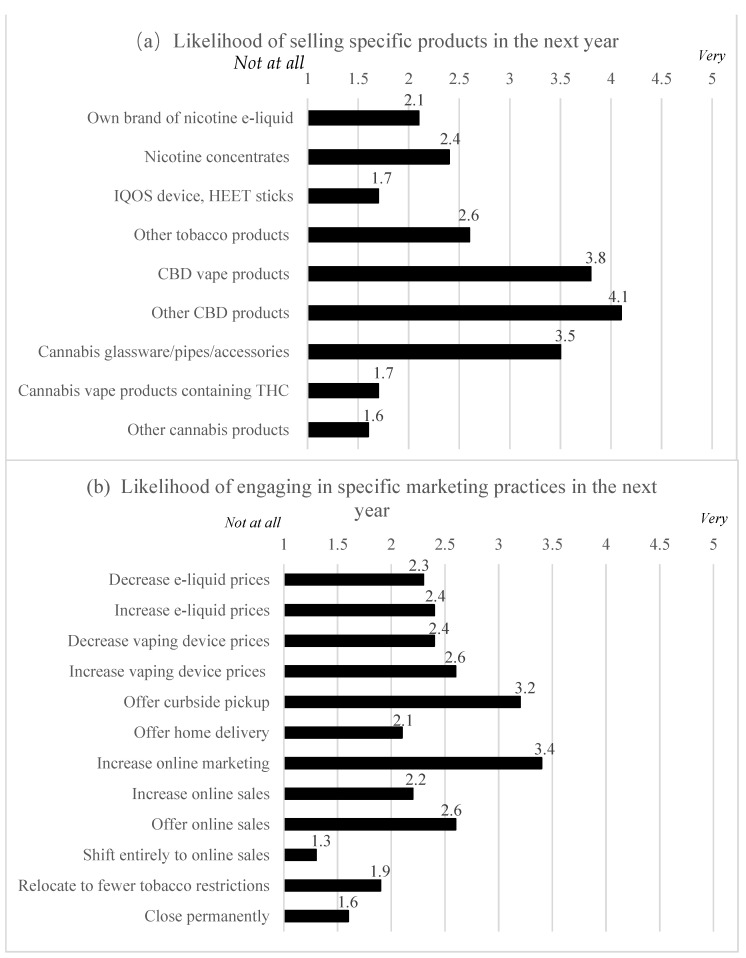
Likelihood of selling specific products and engaging in specific marketing practices in the next year. (Notes: All 2-sample *t*-test *p*-values ≥ 0.05 for vape shops vs. vape-and-smoke shops, except for selling: own brand of nicotine e-liquid (mean (M) = 2.5 vs. M = 1.6, respectively, *p* = 0.027); IQOS device and/or IQOS HEET sticks (M = 1.4 vs. M = 2.0, *p* = 0.040); other tobacco products (M = 1.3 vs. M = 4.1, *p* < 0.001); and glassware, pipes, and accessories for cannabis (M = 2.9 vs. M = 4.1, *p* = 0.013)).

To assess merchants’ *perspectives regarding future retail context*, we asked how likely (11 = “Not at all”, 2 = “A little”, 3 = “Do not Know”, 4 = “Somewhat”, 5 = “Very”) their stores were to offer various products in the next year (e.g., nicotine concentrates, other tobacco products, CBD products; see Figure 1). We also asked: “How do you think your products will change?” (open-ended).

### 2.3. Data Analysis

Using Stata 15.1 (StataCorp, College Station, TX, USA), descriptive analyses were conducted, followed by exploratory bivariate analyses examining differences between vape versus vape-and-smoke shops. As suggested by the description of the measures, the survey was designed to integrate the quantitative and qualitative data, which facilitated analyses. Thematic analysis was conducted to derive common themes from the qualitative, open-ended responses, using both inductive and deductive approaches. Balancing the controversy in qualitative research regarding whether to quantify qualitative results, we chose to indicate the frequency with which themes were provided by participants by categorizing them as “all” (100%), “almost all” (~91–99%), “most” (~76–90%), “the majority” (~51–75%), “some” (~26–50%), and “a few” (~15–25%) [48,49]. We presented representative quotes by MSA and store type (vape vs. vape-and-smoke shop). 

## 3. Results

### 3.1. Participant and Store Characteristics

This sample of 60 owners and managers represented each of the MSAs (Atlanta, *n* = 15.0%; Boston, *n* = 21.7%; Minneapolis, *n* = 20.0%; Oklahoma City, *n* = 23.3%; San Diego, *n* = 6.7%; Seattle, *n* = 13.3%). The majority of participants were male (61.0%), non-Hispanic White (66.7%), and managers (75.0% vs. 25.0% owners). Of these vape retailers, the majority were vape shops (56.7% vs. 43.3% vape-and-smoke shops) and in business in June 2020 (96.6%), and 42.4% were part of a chain.

### 3.2. Current Promotional Strategies and Product Offerings

Overall, 71.7% used social media for promotion, 65.0% had websites, 51.7% offered curbside pickup, and 26.7% offered online sales. In terms of product offerings, 26.7% reported selling their store brand of e-liquid, and 23.3% sold nicotine concentrates. A significant proportion sold other tobacco products (e.g., 36.7% cigars, 30.0% cigarettes, 30.0% roll-your-own tobacco, 25.0% nicotine pouches, 50.0% wrapping papers), while a majority sold CBD products (e.g., 73.3% vape products, 80.0% other products, e.g., lotions, oils, edibles).

### 3.3. Understanding and Implementing Tobacco Regulations 

In response to the question asking whether a state or local T21 policy was in effect prior to the federal T21 policy, 21.7% (*n* = 13) indicated “do not know” (3/9 in Atlanta, 4/13 in Boston, 3/14 in Oklahoma City, 2/4 in San Diego, and 1/8 in Seattle). Of the 47 participants providing other responses, 74.5% (*n* = 35/47) reported that no state or local T21 law applied to their store before the federal T21. Of the 47 responses, 19.1% (*n* = 9/47) were deemed inaccurate: 11.1% (*n* = 1/9) in Atlanta reported a state policy; 30.8% (*n* = 4/13) in Boston reported neither state nor local policy; and 50.0% (*n* = 4/8) in Seattle reported neither state nor local policy. All participants in Oklahoma City and San Diego responded correctly.

In response to the question asking whether state or local sales restrictions on flavored e-cigarettes were in effect prior to the federal ban, 15.0% (*n* = 9) indicated “do not know” (2/9 in Atlanta, 1/13 in Boston, 1/12 in Minneapolis, 3/14 in Oklahoma City, 1/4 in San Diego, and 1/8 in Seattle). Among the 51 participants providing other responses, 78.4% (*n* = 40/51) reported that no state or local restrictions were effective before the federal ban. Of the 51 responses, 27.5% (*n* = 14/51) were deemed inaccurate (albeit with caveats): 61.5% (*n* = 8/13) in Boston indicated that there were no state or local restrictions in place before the federal ban; 25.0% (*n* = 3/12) in Minneapolis indicated state restrictions when only local restrictions applied; 25.0% (*n* = 1/4) in San Diego responded that there were no local restrictions (perhaps as a result of perceived impact of local law); and 12.5% (*n* = 1/8) in Seattle indicated state and local restrictions, which may have been due to the state emergency order that had been temporarily in effect. All participants in Atlanta and Oklahoma City responded correctly.

Participants perceived T21 to be somewhat easy to understand (M = 1.8) and implement (M = 2.0) and perceived the federal flavored e-cigarette ban as somewhat easy to understand (M = 2.3) and implement (M = 2.3; Table 2). Responses to open-ended questions (Table S1) indicated that although the T21 legislation and the federal flavored e-cigarette ban were not difficult to understand, these regulations negatively impacted their business operations and sales (e.g., “We have had to turn away previously legal regular customers who were over 18 but not 21 yet.”—Atlanta vape shop). Some reported experiencing challenges explaining regulations to impacted customers, particularly customers 18–20 years old and flavored product users (e.g., “It was difficult to tell 18–20 year-old customers that had become “regulars” that we could no longer sell to them.”—Oklahoma City vape shop). Some participants indicated that customers tried alternative sources to get the products they need (e.g., shopping at states/localities without regulatory restrictions), used alternative tobacco products (e.g., “Customers who have been impacted instead switched over to devices that allow for flavors.”—Seattle vape-and-smoke shop), or switched back to conventional cigarettes (e.g., “Those who wanted to quit cigarettes wanted flavors, and they ended up going back to cigarettes.”—Seattle vape shop). In addition, some shop merchants questioned the FDA’s intentions regarding T21 legislation and the flavored e-cigarette ban, with a few suspecting that these regulations were instigated by the tobacco industry (e.g., “Anybody that knows anything about the vaping industry is well aware that the success of vaping products is harming the tobacco industry, who will do anything in their power for an extra buck.”—Seattle vape-and-smoke shop).

Moreover, more participants agreed than disagreed that the perceived consequences were severe for violating the federal T21 (M = 3.7; 55.0%, *n* = 33 agreed vs. 13.3%, *n* = 8; 21.7% disagreed, *n* = 13 neutral/did not know) and federal flavored e-cigarette ban (M = 3.6; 48.3%, *n* = 29 agreed vs. 16.7%, *n* = 10 disagreed; 30.0%, *n* = 18 neutral/did not know). While there were no differences in responses between vape vs. vape-and-smoke shop merchants, there was variation by MSA (T21 range: M = 3.3 in Oklahoma City to M = 4.7 in San Diego; flavor ban range: M = 3.3 in Oklahoma City to M = 4.8 in San Diego). Responses to open-ended questions (Table S1) indicated that some reported limited implementation and enforcement (e.g., “A lot of lip service with very little action.”—Minneapolis vape shop); however, the majority mentioned their experiences of regulator inspections from FDA or local authorities or their fears for fines or losing license (e.g., “Our state is very strict, checks on us regularly, and will heavily fine you.”—Seattle vape shop). In addition, a few reported not being affected by the flavored e-cigarette ban because they discontinued selling the impacted products (e.g., “We just don’t carry anything that falls under those restrictions.”—Atlanta vape-and-smoke shop).

### 3.4. Understanding and Implementing COVID-19 Orders

Regarding COVID-related orders, participants perceived regulations to be somewhat easy to understand (M = 2.3) and implement (M = 2.3). Responses to open-ended questions (Table S1) indicated that, although most reported little difficulty understanding or following the COVID-19-related policies, some mentioned the vagueness of regulations (e.g., no clear policies were given, mask compliance is vague, essential business definition is not clear, etc.). In addition, a few reported negative impacts of COVID-19-related regulations on their business (e.g., a decline in sales, prohibited sampling of e-cigarettes indoors, customers refusing to wear masks, curbside delivery service provided to customers not complying with mask mandate, etc.).

### 3.5. Impacts of Tobacco Regulation and COVID-19 on Retail

The impacts of tobacco-related regulations and COVID-19 restrictions on retail were generally neutral, ranging from employee retention (M = 2.4) to the number of usual customers (M = 3.1; Table 2). However, in responses to open-ended questions (Table S1), the majority of participants mentioned that, as a result of T21, they lost underaged customers, particularly those 18–20 years old (e.g., “Customers who were 18–20 were no longer able to purchase items in our store. They were very upset about this, as were we.”—Minneapolis vape shop). In addition, some noted concerns that customers may try other alternatives, including purchasing other flavored tobacco products, switching back to cigarettes, and purchasing at shops not implementing the regulations. In terms of the impacts of the flavored e-cigarette ban, participants mentioned losing customers, as well as customers switching back to conventional tobacco products, being confused about the regulation, and trying other alternatives (i.e., changing products or flavors, purchasing at shops not complying with the regulations).

Participants reported various concerns regarding the negative impacts of these regulations, insufficient evidence bases for regulations, mistrust of the intentions of the flavored e-cigarette ban (e.g., instigated by or to support the tobacco industry), and challenges explaining regulations to affected customers (e.g., those ages 18–20, flavored product customers). Outside of these regulations, some retailers reported concerns about the impacts of taxes on devices, e-liquids, and accessories, the PACT Act, and the FDA’s PMTA processes.

Regarding COVID-19, about half mentioned that vendors responded to the pandemic by offering special promotions (e.g., free masks and hand sanitizers with orders, discounted prices), and some indicated increased communication and/or support (e.g., via newsletters), that their vendors suggested that retailers diversify their product offerings (i.e., to include other tobacco, CBD, etc.), or experienced slow package delivery.

### 3.6. Future Retail Context

The products most likely to be offered in the next year were CBD (vaping and other, M = 3.8 and M = 4.1) and cannabis glassware, pipes, and accessories (M = 3.5; Figure 1). The most likely marketing strategies used in the future were increased online marketing via website or social media (M = 3.4) and offering curbside pickup (M = 3.2). Compared with vape-and-smoke shops, vape shops were more likely to sell their own brand of nicotine e-liquid (M = 2.5 vs. M = 1.6, *p* = 0.027), and less likely to sell IQOS device and/or IQOS HEET sticks (M = 1.4 vs. M = 2.0, *p* = 0.040), other tobacco products (M = 1.3 vs. M = 4.1, *p* < 0.001), and glassware, pipes, and accessories for cannabis (M = 2.9 vs. M = 4.1, *p* = 0.013).

Qualitative responses (Table S1) indicated that stores planned to sell disposable e-cigarettes, and a few also mentioned nicotine-free devices and tobacco-free nicotine, depending on the FDA’s future regulations on nicotine levels and customers’ needs. In addition, the majority indicated plans to offer CBD products and accessories, with some also talking about the prospects of increasing their offerings of kratom (i.e., tropical tree leaves that contain compounds with psychotropic effects) and delta-8-THC (i.e., which produces euphoric effects similar to but milder than those of delta-9-THC, the well-known psychoactive compound in cannabis).

## 4. Discussion

These data from a 2021 survey of 60 vape and vape-and-smoke shop owners and managers in six US MSAs indicated that federal policies and COVID-19-related orders were perceived to be somewhat easy to understand and implement, despite prior findings indicating noncompliance [23,32,33], and current findings indicating limited knowledge of relevant state and/or local T21 and flavored e-cigarette sales restrictions. Moreover, in response to increasing restrictions on e-cigarettes, vape retailers are expanding their product offerings, particularly to CBD products (as noted previously [32,34,43]), and are also increasing their online marketing, which reflects a global trend toward online e-cigarette marketing and sales [4,5]. These findings are critical to informing future surveillance of the e-cigarette retail environment, consumer behavior, and regulatory compliance.

With regard to regulatory knowledge and implementation, approximately 40% of merchants either did not know or inaccurately reported whether state or local T21 laws or flavored e-cigarette sales restrictions were in effect before the respective federal policies. This undermines their accounts that the federal T21 and flavored e-cigarette ban have been somewhat easy to understand and implement, as well as prior accounts that a key part of vape retailers’ personnel training focuses on tobacco regulations and compliance [44]. Notably, participants indicated little support for implementing regulations, underscoring the need for assistance from regulatory agencies and increased efforts to disseminate related information as these policies go into effect.

Participants noted relatively little impact of tobacco regulations or COVID-19-related orders on their overall sales or retail activity which, could be in part due to relatively low compliance with COVID-19-related state orders among vape shops [32]. Additionally, 38% of this sample were located in Atlanta or Oklahoma City, where business closures either did not apply (Georgia) or were in place only briefly (Oklahoma) [32].

Moreover, a majority of participants reported the consequences were severe for violating the tobacco regulations, with just over a quarter reporting being neutral/not knowing. Importantly, participants in Oklahoma City perceived the least severe consequences while those in San Diego perceived the most severe, which parallels the actual consequences in terms of fines for violating underage sales laws (i.e., roughly five times greater fines in California vs. Oklahoma) [50,51]. In addition, most participants mentioned their experiences with inspections by the FDA or local authorities, or their fears of fines or losing their retail license. With this in mind, it is critical to consider merchants’ motivation to comply with regulations, as compliance is necessary for regulations to achieve their intended effects.

Despite survey data indicating relatively few impacts (on average), participants’ open-ended comments noted a variety of concerns regarding the negative impacts of these regulations (e.g., financial), the insufficient evidence base for these regulations, and concerns that restrictions on the e-cigarette industry are instigated by the tobacco industry, which have been raised as vape retailers’ concerns in prior research [41,42]. Moreover, merchants expressed concerns regarding the negative impacts of T21 and flavored e-cigarette restrictions on their customers, noting that customers may purchase other flavored products, switch back to cigarettes, and/or purchase products at shops not complying with the regulations. These concerns are consistent with research indicating that e-cigarette customers may attempt to obtain banned tobacco products through alternate sources [52,53], and/or substitute other flavored products [53,54]. Thus, it is key to examine both retailer and consumer compliance with tobacco regulations, particularly to determine the extent to which such efforts yield the desired outcomes—and if not, how regulations must address contributing factors in the future.

Merchants’ perspectives regarding the implications of tobacco regulations provided valuable insights into shifts in the e-cigarette marketplace that will likely occur. In this sample of retailers, roughly half owned or managed vape shops that do not sell combustible tobacco products [6] and may advocate vaping to aid in cigarette cessation, despite such marketing being prohibited [38,55]. However, as a result of such regulations, many vape shops have faced—or are beginning to face—a difficult decision: struggle to survive, close, or rebrand with new inventory—which likely includes a wider variety of tobacco products [34,42,43,56]. Consistent with previous studies [34,42,43], merchants indicated that CBD and related paraphernalia is prominent within the vape retail setting, with 73.3% selling CBD vape products and 80.0% selling other CBD products. Moreover, participants reported that CBD would continue to be an important component of their product offerings, likely reflecting the rapid expansion of the CBD market (including products administered similarly to tobacco products, e.g., vaped or smoked) [57,58,59]. Roughly one-tenth of our sample reported selling vape products containing THC, and about one-sixth intended to sell delta-8-THC and/or kratom products in the future. These stores were in all six MSAs except Seattle, where delta-8-THC products were illegal to sell [60]. Despite retailers’ interest in CBD and THC products, it is important to note that the federal government [61,62,63] and many states (e.g., California [64]) are advancing agendas to regulate CBD products. In addition, e-cigarette and vaping-associated lung injuries (EVALI) raised consumer awareness about adverse health effects of vaping e-liquids that contain THC [65] which may reduce demand for such products and/or increase regulatory oversight (e.g., mandating warnings as in Massachusetts [66]). Future research is warranted to examine how the vape retail environment may adapt or evolve as the regulation of nicotine and legalization and regulation of cannabis and cannabis-derived products increases.

In addition, findings indicated that a critical component of the marketing strategy among vape retailers is the online environment, noted as being the fastest-growing vape retail segment [4,5]. In this sample, the majority of participants reported maintaining a store website (65.0%) and/or social media (71.7%) for online promotion [32,67], and 26.7% offered online sales. While only 11.7% offered home delivery, 51.7% reported offering curbside pickup, largely as a result of COVID-19 and related state orders [32]. Participants reported that online promotion and curbside delivery were the most likely to continue in the future, while online sales and home delivery were less likely to be used, perhaps due to the PACT Act and its implications for delivery [14].

The current findings highlight the complexities in implementing and enforcing tobacco-related regulations and COVID-19-related orders among vape and vape-and-smoke shops and the importance of continued surveillance in assessing the impact of government regulations on the vape and tobacco retail market. The results also suggest that public education for retailers and consumers by government entities may be needed to reduce confusion and improve policy implementation and enforcement [11]. Furthermore, the findings on likely future retail activities may provide preliminary evidence to support future regulatory efforts to reduce underage access to tobacco and CBD products.

Study limitations include the use of a small, non-probability sample with limited generalizability to these MSAs and other US states. Relatedly, this study is largely descriptive by design, as this small sample size precluded the ability to conduct more sophisticated analyses. Nonetheless, these findings provide insights that can be further investigated in studies of vape retail with larger sample sizes and/or using other approaches. Additionally, study assessments, which were adapted from prior research [23,35,47], were brief, largely single-item measures (to reduce participant burden); moreover, assessments relied on self-report and are subject to recall and social desirability bias. The qualitative data was limited in depth of response and, in some cases, clarity of intended meaning. Finally, this study focused on T21, flavored e-cigarette bans, and COVID-19-related orders and did not include questions regarding the implications of other existing laws (e.g., the PACT Act [14]) or proposed legislation (e.g., increasing federal tobacco taxes).

## 5. Conclusions

Owners and managers of vape and vape-and-smoke shops demonstrated limited knowledge of relevant state/local T21 and flavored e-cigarette sales restrictions. Although retailers perceived federal policies and COVID-19-related orders to be somewhat easy to understand and implement, they voiced concerns regarding the negative implications of regulation on business, industry, and consumers. Notably, CBD products and online marketing have been and will continue to be important aspects of the vape retail environment. Together, these represent significant concerns for regulatory compliance potentially due to limited knowledge, motivation to comply, or both as well as the need for resources to improve compliance with federal, state, and local policies, and to anticipate how the marketplace may adapt and evolve.

## Figures and Tables

**Table 1:** Promotional activities and product offerings in a sample of vape and vape-and-smoke shop owners or managers, *n* = 60.

Variable	*n*	%
***Promotional activities***		
Uses social media	43	71.7%
Has a website	39	65.0%
Offers curbside pickup	31	51.7%
Offers online sales	16	26.7%
Offers home delivery	7	11.7%
None of the above	6	10.0%
***Product offerings***		
Tobacco-related products ^1^		
Your own brand of nicotine e-liquid	16	26.7%
Nicotine concentrates to add zero-nicotine flavored e-liquid	14	23.3%
Cigars	22	36.7%
Cigarettes	18	30.0%
Shish tobacco used in hookah pipes	18	30.0%
Roll-your-own tobacco	18	30.0%
Nicotine pouches	15	25.0%
IQOS device and/or IQOS HEET sticks	3	5.0%
Wrapping papers	30	50.0%
Hookah pipes	20	33.3%
None of the above	13	21.7%
CBD or cannabis products ^1^		
CBD vape products	44	73.3%
Other CBD products	48	80.0%
Glassware, pipes, and accessories for cannabis	27	45.0%
Cannabis vape products that contain THC	7	11.7%
Other cannabis products	5	8.3%
None of the above	4	6.7%
Other products ^1^		
Beverages	24	40.0%
Food	10	16.7%
Apparel/merchandise with store brand	21	35.0%
Apparel/merchandise branded with a product line	11	18.3%
None of the above	19	31.7%

^1^ “Other” responses: *n* = 14; *n* = 9; *n* = 1.

**Table 2:** Perceptions of tobacco regulations and COVID-19 orders on vape retail among vape and vape-and-smoke shop owners or managers, *n* = 60.

Variable	*n*	%
State or local T21 policy before federal T21 (per retailer report)		
State	11	18.3%
Local	1	1.7%
Neither state nor local	35	58.3%
Do not know	13	21.7%
Incorrect response among those not reporting “do not know”	9	19.1%
State or local e-cigarette flavor ban before federal flavor ban (per retailer report)		
State	9	15.0%
Local	2	3.3%
Neither state nor local	40	66.7%
Do not know	9	15.0%
Incorrect response among those not reporting “do not know”	14	27.5%
Perceived difficulty: *1 = very easy to 5 = very difficult*	**Mean**	**SD**
Understand the federal T21 legislation	1.8	1.4
Follow the federal T21 legislation	2.0	1.5
Understand the federal flavor product restrictions	2.3	1.6
Follow the federal flavor product restrictions	2.3	1.5
Perceived consequences of noncompliance: *1 = strongly disagree to 5 = strongly agree*		
The consequences for violating the T21 policy are severe	3.7	1.3
The consequences for violating flavored e-cigarettes ban are severe	3.6	1.3
During COVID-related restrictions, perceived difficulty: *1 = very easy to 5 = very difficult*		
Understand the COVID-19-related restrictions on business operations	2.3	1.5
Follow the COVID-19-related restrictions on business operations	2.3	1.4
Impact of tobacco regulations and COVID-19 on: *1 = decreased a lot to 5 = increased a lot*		
Vaping device sales	3.0	1.4
E-liquid sales	2.8	1.4
Vaping device prices	2.9	1.0
E-liquid prices	2.9	1.0
Number of device types offered	3.0	1.4
Number of e-liquid flavors/types offered	2.9	1.3
Number of customers who are frequent patrons (“usuals”)	3.1	1.4
Number of customers that are not frequent patrons	3.0	1.2
Employee retention, or staffing levels	2.4	1.1
Online sales	2.8	1.2

## Data Availability

Data not publicly available (available upon request).

## Supplementary Materials

The following supporting information can be downloaded at: https://www.mdpi.com/article/10.3390/ijerph19073855/s1, Table S1: Themes and quotes of responses to open-ended questions among vape and vape-and-smoke shop owners or managers, *n* = 60.
