# Supplementary material for: The Impact of Recent Tobacco Regulations and COVID-19 Restrictions and Implications for Future E-Cigarette Retail: Perspectives from Vape and Vape-and-Smoke Shop Merchants
Source: Int J Environ Res Public Health. 2022 Mar 24;19(7):3855. doi: 10.3390/ijerph19073855 (PMC8997836; doi:10.3390/ijerph19073855)
Supplement: Supplementary file 1 [file ijerph-19-03855-s001.zip › ijerph-1604865-supplementary.pdf]

**Supplementary Table S1. Themes and quotes of responses to open-ended questions among vape and vape-and-smoke shop owners or managers, N=60.**

| Themes | Quotes |
|---|---|
| **What was or has been difficult about understanding and/or following the T21 legislation? The flavored product bans?** | |
| Business operations | – It was difficult to tell 18-20 year-old customers that had become "regulars" that we could no longer sell to them. – *Oklahoma City vape shop*<br>– Not hard to understand or follow; it's just hard on the sales! – *Boston vape-and-smoke shop*<br>– When military kept coming in to say they were 18 with a military ID, we had to say no because laws changed again. – *San Diego vape-and-smoke shop* |
| Questioning FDA's purpose | – Anybody that knows anything about the vaping industry is well aware that the success of vaping products is harming the tobacco industry, who will do anything in their power for an extra buck. – *Seattle vape-and-smoke shop*<br>– Just why the FDA thinks or feels it is necessary or helpful in their end game. It is counter-productive if you ask us. – *Oklahoma City vape shop*<br>– The procedures have been straightforward, but I question the government's motives in doing so. One is able to serve in the military and star in an adult film, but cannot buy vape products. Either one is an adult, or not. – *Atlanta vape shop* |
| Not difficult to understand or comply | – There has been nothing difficult about understanding and/or following the T21 legislation or the flavored products ban. We put up signs stating the T21 legislation and explained to customer when asked. – *Oklahoma City vape shop*<br>– Nothing, our corporate office made it very clear what the legislations were. – *Atlanta vape-and-smoke shop*<br>– Nothing really, under 21 you shouldn't come here, I check their ID. – *Boston vape shop* |
| Vagueness | – No information was provided to us. – *Seattle vape-and-smoke shop* |
| **What was or has been difficult about understanding and/or following the COVID-related policies, if anything?** | |
| Impact on business | – It's not hard to understand; it's just hard to deal with; business has been down almost 40%. – *Boston vape-and-smoke shop*<br>– Not allowing customers to vape indoors and "try before they buy". – *Seattle vape shop*<br>– That costumers don't like to comply with the mask mandate for businesses. – *Minneapolis vape-and-smoke shop*<br>– The fact that the government can tell people and businesses that they are "non-essential". Every person, every job, and every business is essential to someone. – *Minneapolis vape-and smoke shop* |
| Vagueness | – No clear policies were given. – *Oklahoma City vape shop*<br>– Mask compliance is kind of vague. – *Boston vape shop*<br>– The information hardly if ever reaches us, and we are left in the dark. – *San Diego vape-and-smoke shop* |
| Not difficult to understand or comply | – Nothing has been difficult. We require all customers to wear a mask inside the store until the CDC recommends otherwise. For those who are unable or unwilling to wear one, and to comply with ADA, we offer curbside/car side delivery. – *Atlanta vape shop* |
| **In your experience, what is being done to enforce or oversee compliance with the e-cigarette flavor restrictions, if anything? What do you perceive as the consequences or risks of not being compliant, if anything?** | |
| Limited implementation and enforcement | – A lot of lip service with very little action. – *Minneapolis vape shop*<br>– During my shifts, we've never had a compliance check, although COVID interrupted compliance checks in general. – *Seattle vape-and-smoke shop* |

| | |
|---|---|
| | – Enforcement or oversee compliance is nonexistent in our area. You can report a store selling to underage customers and nothing is done. I personally don't see any consequence of not being compliant. – *Oklahoma City vape shop* |
| Regular inspections from FDA or local authorities | – Every municipality's health department, the state's health department (plus the FEDS) by law have to sting us quarterly. One shop was fined $8,000 last week for selling flavored e-juice to an underaged kid. That's 2 strikes at once. The store will lose its license with the next mistake in the next 24 months (state law) and possibly worse depending on the town/city in which they operate. – *Boston vape shop*<br>– FDA does yearly inspections. I'm not sure what the consequences are beyond closing down, but I'm sure they aren't pleasant. – *Atlanta vape shop*<br>– Our state is very strict, checks on us regularly, and will heavily fine you. – *Seattle vape shop*<br>– The FDA is checking online presence for advertising sales/carrying stock of illegal e-cigarette flavors. They are sending warning letters to noncompliant companies. I think they are fining or closing noncompliant businesses. – *Oklahoma City vape shop*<br>– The government has handed enforcement of the PACT act over to the ATF. – *Seattle vape shop* |
| Fines or loss of license as a deterrent | – The crazy fines are about all you need to enforce it. They are so large, some small local vape stores would shut down after one fine. – *Boston vape shop*<br>– Main risk is losing tobacco license, shutting down business. – *Boston vape-and-smoke shop* |
| No impact | – I do not carry the restricted products in any flavor, even the ones allowed. I think I could be shut down. – *Oklahoma City vape shop*<br>– We just don't carry anything that falls under those restrictions. – *Atlanta vape-and-smoke shop* |
| **How have your customers been impacted by the federal T21 legislation?** | |
| Lost underaged customers | – Customers who were 18-20 were no longer able to purchase items in our store. They were very upset about this, as were we. – *Minneapolis vape shop*<br>– We have had to turn away previously legal regular customers who were over 18 but not 21 yet. – *Atlanta vape shop*<br>– We lost a few customers because of the few restrictions but overall not that bad. – *Oklahoma City vape shop*<br>– Yes, our sales have gone down since the T21 legislation. – *Minneapolis vape shop* |
| Consumers using alternative products or product sources | – Forced to overpay for juice they don't really want. – *Boston vape shop*<br>– The customers that were 18 and then had to stop was quite unfair and probably started smoking cigarettes. – *Atlanta vape shop*<br>– They just go to the Indian reservation down the street where the regulations don't apply. – *Seattle vape shop* |
| Merchant and consumer confusion about T21 | – No, it does not apply in our state. – *Oklahoma City vape shop*<br>– There was a bit of confusion amongst customers when the policy was initially enacted. – *Seattle vape-and-smoke shop* |
| No impact or helpful | – It has controlled customers from coming in that are younger ages. It has helped. – *Seattle vape-and-smoke shop* |
| **How have your customers been impacted by the flavored e-cigarette product sales restrictions?** | |
| Lost consumers, stopped selling certain products | – We lost some customers when Juul flavors were banned. – *Oklahoma City vape shop*<br>– We have stopped carrying certain products, such as Juul. – *Atlanta vape shop* |
| Consumers switched to conventional cigarettes | – Severely, we lost a lot of new customers because of the policy. Some come back and just grab the tobacco but it's been pretty bad. – *Boston vape-and-smoke shop* |

| | |
|---|---|
| | – A lot of customers struggled, especially ones who liked flavors. Those who wanted to quit cigarettes wanted flavors, and they ended up going back to cigarettes. – *Seattle vape shop* |
| Consumers using alternative products or product sources | – Changed to different vape products. – *Oklahoma City vape shop*<br>– Customers who have been impacted instead switched over to devices that allow for flavors – *Seattle vape-and-smoke shop*<br>– Forced to overpay for juice they don't really want. – *Boston vape shop*<br>– They choose to go to the Indian res and avoid rules. – *Seattle vape shop*<br>– They go to New Hampshire. – *Boston vape shop* |
| No impact | – Our customers really have not seen much of a change, as our local and state government has yet to implement a flavor ban. – *Atlanta vape shop*<br>– Some have been upset but understand that we are following federal law. However, there are stores in our vicinity that do not follow the "restrictions" and continue to sell products to customers. – *Oklahoma City vape shop* |
| **How have your vendors responded to COVID-19, if at all? For example, did they provide resources to help you understand/follow the regulations? Suggest new ways to market and sell your products during the restrictions? Other responses?** | |
| Limited information | – They probably did, but we already enact pretty strict policies regarding COVID. I they provided resources, I didn't pay it any mind. – *Seattle vape-and-smoke shop* |
| Provided promotions | – Yes, vendors have been including free masks with orders as well as hand sanitizers. – *Seattle vape shop*<br>– Many vendors offered their products at deep discount before going out of business. – *Atlanta vape shop*<br>– They allow extra wait times for packages to "quarantine" before sending them to us, as well as most of them include free face masks and bottles of hand sanitizers for our employees to use. – *Atlanta vape shop*<br>– Vendors suggested to provide us with marketing materials. – *San Diego vape-and-smoke shop* |
| Suggested changes in product offerings | – They simply suggest becoming a head shop, CBD, Kratom and Delta 8 store. While we sell all of them, CVS and Walmart sell CBD (and don't understand the products), Kratom and Delta 8 are the next to be regulated by the FEDS. Both won't make it in the US. – *Boston vape shop*<br>– They did not, product stock suffered greatly. – *San Diego vape-and-smoke shop* |
| Changes in processes | – It is mainly a much slower process, and many things are hard to get. – *Atlanta vape shop*<br>– They moved out of state and send emails on all new regulations. – *Seattle vape shop* |
| **Outside of T21 and flavor bans, what other tobacco-related regulations are impacting your shop – or do you think will impact your shop in the future?** | |
| Tax on vaping devices, e-liquids, and accessories | – Expanding in this industry in the Twin Cities will be difficult because there are so many cities with flavor bans and similar regulations. Minnesota is also trying to tax devices the same as any other tobacco product. We have a 95% tax on tobacco. This will very negatively affect business because for that increase in price a good number of people will just drive out of state to purchase a new device. That has been fairly common practice for Minnesotans who buy cigarettes and dip in bulk so I would expect the trend to follow with vaping if they continue to over tax us. – *Minneapolis vape shop*<br>– The proliferation of new and expanded taxes will have a major impact on not only our store but all other tobacco shops in the future. – *Minneapolis vape-and-smoke shop*<br>– Main regulation is new Massachusetts excise tax. Will push prices up for customers. – *Boston vape-and-smoke shop* |

| | |
|---|---|
| PACT Act | – The PACT ACT is a shipping regulation that severely impacted the industry as well as our shop. It made it extremely difficult to receive products, and made our shipping times have at least doubled if not tripled from what they used to be. – *Atlanta vape shop*<br>– The new PACT act is affecting the industry by limiting how we can have nicotine products delivered, and also put CBD in the same category as nicotine. We have shifted to deal with it, but it's another obstacle we had to overcome. – *Minneapolis vape-and-smoke shop*<br>– Wholesale vendors are having problems in shipping, even to vape shops. – *Oklahoma City vape shop* |
| FDA regulations (e.g., prohibited sampling) | – FDA PMTA regulations. – *Minneapolis vape shop*<br>– Customers want to know how a flavor tastes first before buying, but we don't offer sampling anymore due to regulations. – *San Diego vape-and-smoke shop* |
| Online sales ban | – Because of the ban of online sales, our foot traffic has increased. – *Oklahoma City vape shop* |
| **How do you think your products will change, if at all? For example, what new products (e-liquids, devices, others) do you expect to be important, and what products do you anticipate being less important or discontinuing? Why?** | |
| Disposable e-cigarettes | – I believe that disposable vapes, which have made a HUGE resurgence over the last 2 years, will be impacted the most. – *Seattle vape shop*<br>– Disposables and nic salts on the rise. High wattage devices on low. – *Minneapolis vape shop*<br>– Disposable vapor products are very important right now. They are between getting banned from the FDA & sales right now. They are convenient and have sweet and fruity flavors. I'm sure they will become illegal. That will greatly impact our sales. – *Oklahoma City vape shop* |
| Nicotine-free device | – The industry will either go to zero-nicotine juices with a nicotine additive as they have in the UK/EU, or there will be a complete ban. – *Atlanta vape shop*<br>– I think, in the near future, there will be some sort of nicotine cap or maximum amount they will allow to be in e-liquid, meaning that "salt nicotine" devices and refillable pod systems will phase out. – *Atlanta vape shop* |
| Tobacco-free nicotine | – The vaping industry will be using tobacco free nicotine. – *Oklahoma City vape shop* |
| CBD products | – Think they will continue what they currently have. May add more CBD products. – *Boston vape shop*<br>– We are bringing in new devices geared towards CBD use. – *Minneapolis vape shop*<br>– With the rise of DELTA 8 and now DELTA 10 products, I see things shifting more toward those products and less CBD products. I don't think we'll completely discontinue CBD, but we're definitely not emphasizing it anymore. – *Minneapolis vape-and-smoke shop* |
| Evolving depending on government regulations | – Over time, I believe that products will change based on the guidelines from the federal government. Some products will remain on the market while others will go away based on supply and demand along with what the consumer wants. – *Oklahoma City vape shop* |
